# The impact of warfarin on operative delay and 1-year mortality in elderly patients with hip fracture: a retrospective observational study

**DOI:** 10.1186/s13018-019-1199-5

**Published:** 2019-06-04

**Authors:** Gaetano Caruso, Mattia Andreotti, Tedi Marko, Francesco Tonon, Nicola Corradi, Damiano Rizzato, Alessandra Valentini, Giorgia Valpiani, Leo Massari

**Affiliations:** 1 Department of Biomedical and Speciality Surgical Sciences University of Ferrara, Via Luigi Borsari 46, 44121 Ferrara, Italy; 2 Orthopaedic and Traumatology Unit, Sant’Anna University Hospital of Ferrara, Via Aldo Moro 8, 44124 Cona, Ferrara Italy; 3 Department of Morphology, Surgery and Experimental Medicine University of Ferrara, Via Luigi Borsari 46, 44121 Ferrara, Italy; 4 Student in Statistical Sciences at University of Bologna, Via Zamboni 33, 40126 Bologna, Italy; 5 Research and Innovation Office, Sant’Anna University Hospital of Ferrara, Via Aldo Moro 8, 44124 Cona, Ferrara Italy

**Keywords:** Proximal femoral fractures, Elderly patients, Warfarin, Surgical timing, Mortality

## Abstract

**Background:**

Guidelines underline the importance of early surgery in elderly patients with proximal femoral fractures. However, most of these patients present a high number of comorbidities, some of which require the use of warfarin. Waiting for INR decrease is a cause of surgical delay, and this influences negatively their outcome.

**Methods:**

We retrospectively reviewed all patients with proximal femoral fracture admitted to our unit from March 2013 to March 2017 to determine whether warfarin therapy is associated with reduction of survival, delay of surgery, and increased blood loss. From 1706 patient, a total of 1292 fulfilled the eligibility criteria and were included. Data regarding general information (type of fracture according to AO/OTA classification), pharmacological history regarding anticoagulant therapy pre-admission, surgery (type of surgery and time to surgery), clinical findings (blood loss), and date of exitus were collected.

**Results:**

We identified 157 patients with warfarin, 442 with antiplatelet agents (aspirin, clopidogrel, ticlopidin), and 693 in the control group. We observed a significant difference in the warfarin group regarding an increased ASA score, Charlson Comorbidity Index, and blood loss. Patients taking warfarin experience delay to the theater significantly more than the other groups. Patients in warfarin therapy have a 42% higher risk of death within 1 year from their surgery. Patients who underwent surgery after 48 h have 1.5 times higher risk of mortality with respect to the patients who underwent surgery within 48 h.

**Conclusion:**

Warfarin therapy at the time of proximal femoral fractures is associated with increased time to surgery, blood loss, and mortality.

## Introduction

Hip fractures are common orthopedic injury and are a major cause of morbidity and mortality in the elderly. One-year mortality after hip fracture ranges from 14 to 36% [1–3]. Delayed surgical treatment and pre-admission comorbidities are associated with an increased post-operative mortality [4, 5]. Current guidelines recommend surgery within 48 h [6]. However, the association between delayed surgery and mortality remains elusive because of strong confounding by comorbidity factors. Long-term anticoagulant treatment is frequently encountered in patients with proximal femoral fracture (PFF) and associated with surgical delay [7]. Despite this, there are no set national standards for management of drug-induced coagulopathy pre-operatively in the context of hip fractures.

Warfarin is the most widely prescribed anticoagulant and it has been estimated that 4–8% of these patients are in therapy with it in the UK [8]. Traditionally, warfarinised patients were considered high-risk patients due to increased bleeding and associated perioperative complications, and their management could cause surgical delays. The current clinical practice recommends to postpone surgery until a patient’s international normalized ratio (INR) decreases below 1.5. The time taken to achieve a safe INR together with the presence of multiple comorbidities has the potential to delay surgical treatment [9]. One of the strategies to reduce the bleeding in these patients is to temporarily postpone the warfarin administration and then wait until the anticoagulant effect of the warfarin has subsided, the action of warfarin lies between 2 and 5 days [10].The use of vitamin K to reverse the effect of warfarin has been shown to be safe and effective in the context of PFF surgery even though there is no widely accepted consensus on the absolute indications, dosage, and route of vitamin K treatment in the context of hip fracture [11].

The role of the antiplatelet therapy (aspirin, clopidogrel, ticlopidin) in the management of elderly patients with hip fractures seems to be less relevant. A clinical study addressing the use of both aspirin and clopidogrel when surgery for hip fractures is not delayed concluded that there was no difference in bleeding complication, blood loss, or transfusion requirements, when compared with those not on antiplatelets [12].

More recently, the factor Xa inhibitors have been popularized (apixaban, dabigatran, and rivaroxaban) [13]. Common practice is to interrupt the factor Xa inhibitors for 24–48 h and undertake surgery thereafter [14]. Patients with PFF taking Xa inhibitors seem to have a longer delay in surgery compared to patients on warfarin or no anticoagulation [15].

The purpose of this study is to determine whether warfarin therapy at the time of admission for PFF is associated with reduction of survival, delay of surgery, and increased blood loss.

## Materials and methods

This is a retrospective cross-sectional study on consecutive patients with PFF admitted between March 2013 and March 2017 at our Orthopedic and Traumatology Unit located in the northeast of Italy (catchment area which caters to roughly 350,000 inhabitants).

**Fig. 1:**
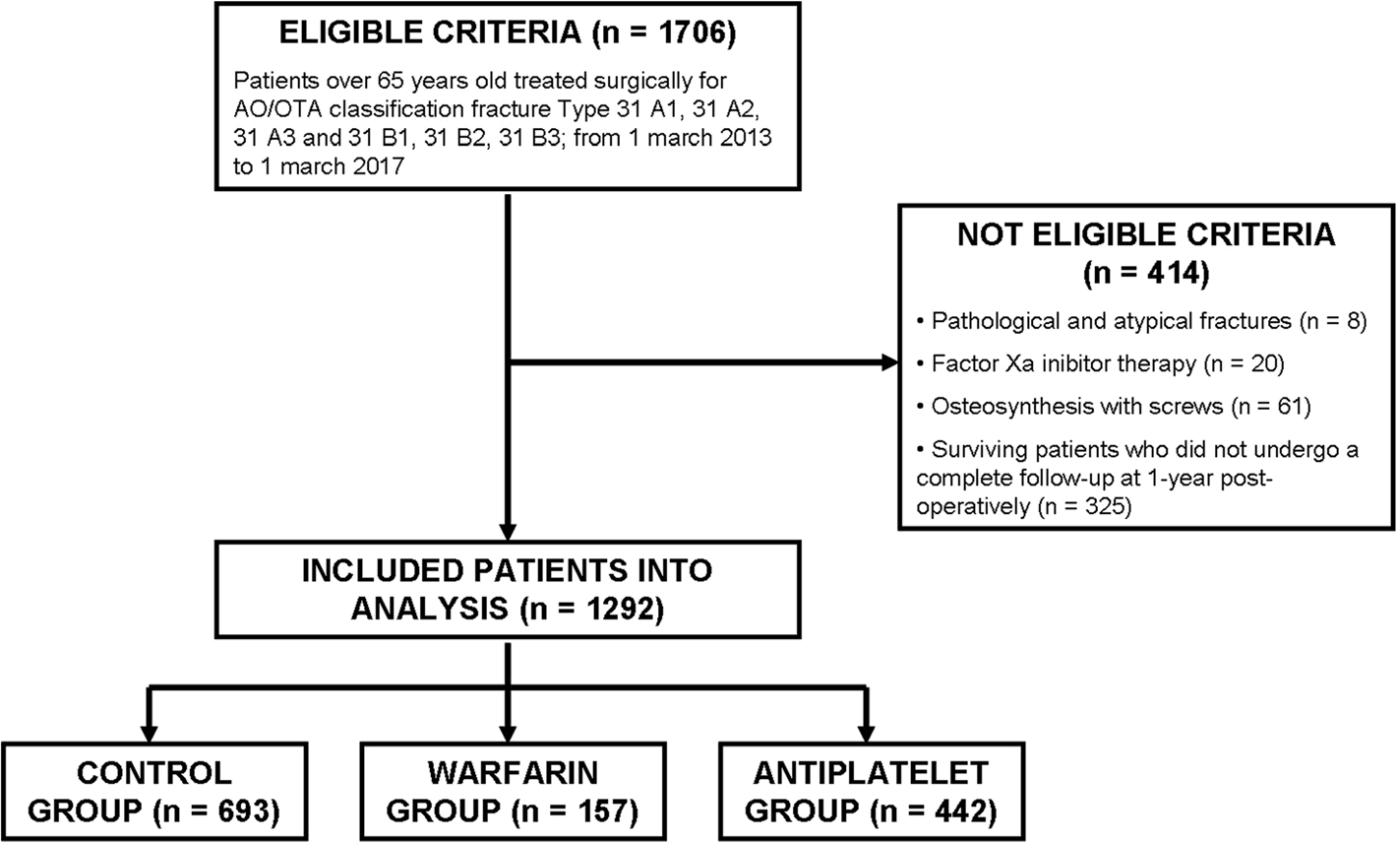
Flowchart describing the inclusion and exclusion criteria of the study

Patients older than 65-years undergoing surgery for PFF were identified retrospectively from a hospital discharge database. The fractures were classified according to the AO-Müller/Orthopaedic Trauma Association fracture and dislocation classification (AO/OTA) [16]. The inclusion criteria were to have isolated proximal femoral fracture 31-A1, 31-A2, 31-A3, 31-B1, 31-B2, and 31-B3 (according to AO/OTA classification) and to have been treated surgically with joint reconstruction (total or emi- hip arthroplasty) or fixation with intra- or extramedullary devices (proximal femoral plate or proximal femoral short and long nail) with a minimum follow-up of 12 months. Patients with pathological or atypical femoral fractures, or fractures treated with other fixation devices (fixation with screws); patients with factor Xa inhibitors therapy (apixaban, rivaroxaban, fondaparinux); and patients still alive that did not undergo a complete follow-up at 365 days post-operatively were excluded (Fig. 1).

Data regarding general information, pharmacological history, surgery, date of exitus, and clinical findings were collected. We calculated hours between admission and surgery for all patients using threshold of 48 h as suggested in literature [6]. The American Society of Anesthesiologists Physical Status Classification System score (ASA) and Charlson Comorbity Index (CCI) were extracted to evaluate patient comorbidities [17]. In order to estimate blood loss, we used the method of hemoglobin balance described by Foss and Kehlet. [18] We gathered preoperative and 3 days post-operative hemoglobin values and units of fresh packed red blood cell transfused, as this method expected. We registered mortality data at 30, 180, and 365 days for each group of patients.

In pharmacological data, we divided the whole sample in three groups based on the anticoagulant therapy that they took at the admission: warfarin, antiplatelet drugs (aspirin, clopidogrel, ticlopidine), and the control group contained patients that did not take any anticoagulant or antiplatelet agents. Patients on warfarin therapy underwent a discontinuation protocol as suggested by the British Society of Haemetalogy [19] that consists of using oral or intravenous vitamin K in order reduce the INR values to operate these patients within 48 h. Patients were not treated with a standardized warfarin reversal protocol and received intravenous administration of a dose of 10 mg/ml of vitamin K1 (Konakion) maximum twice a day based on the INR values. INR values were checked at the admission of the patient and every 8 h until surgery. We also shared our groups in those whom underwent surgery within or after 48 h, as well as by type of surgery to evaluate the potential association between warfarin treatment and time to surgery and surgical delay and mortality.

### Statistical analysis

Continuous data were checked for normality using Shapiro–Wilk test. All continuous variables were expressed as means and standard deviation (SD) for normality or medians and interquartile ranges (IQR25–75) for non-normally distributed continuous variables. Categorical data were expressed as total numbers and percentages. Statistical comparisons of continuous variables were performed using unpaired analysis of variance for normally distributed and nonparametric Kruskal–Wallis test for non-normally distributed variables, whereas categorical variables were assessed using Pearson’s *χ*^2^ test. Survival curves estimated with Kaplan–Meier methods were compared via log-rank test among groups of anticoagulant therapy. In this case, mortality was assessed at 30 days, 180 days, and 365 days. Kaplan–Meier survival estimation model was performed for long-term survival up to 1-year follow-up comparing the impact of three different groups. Age, gender, age, anticoagulant therapy, ASA score, Charlson Comorbidity Index, AO/OTA Classification, types of surgical procedure, time to surgery, and blood loss were assessed as a prognostic factor for survival using Cox regression analysis [20, 21]. Additionally, a stepwise selection procedure based on likelihood-ratio test was performed. In backward elimination, we considered a removal probability equal to 0.10. We analyze Schoenfeld residuals to test the assumption of proportional hazards [22]. For the time to event analysis, patients were censored at the time of death. Further, we calculated multivariate Cox regression analysis adjusted for potential confounders to assess which parameters had an independent association with mortality. Goodness of fit of the model is evaluated graphically by Cox–Snell residues. A two-sided *p* value < 0.05 was considered statistically significant. Statistical analysis was performed using STATA 12.1 (StataCorp, TX, USA).

## Results

From 1706 patients admitted with hip fractures during the study period, a total of 1292 (76%) patients fulfilled eligibility criteria and were included in the study, 414 (24%) patients were excluded. We identified 157 (12%) patients with warfarin, 442 (34%) with antiplatelet agents, and 693 (54%) in the control group. All demographics and clinical data of each group are summarized in Table 1. Comparing the data of the 3 groups (warfarin, antiplatelet, and control group), we noticed that the sample is composed by prevalently female patients (74%) and there were no significant differences regarding age at the time of surgery. Half of the patients (*n* = 641) belong to the highest ASA risk classes (ASA 4/5). ASA score and Charlson Comorbidity Index were significantly higher in the warfarin group (*p* < 0.001). Regarding the time of surgery, 54% of the patients in the warfarin group underwent surgery after 48 h, while the 63% of the patient in the antiplatelet group and the 69% of the patient in the control group underwent surgery within 48 h, showing an opposite trend (*p* < 0.001). The median blood loss was significantly higher in the warfarin group (*p* = 0.0054). Mortality was higher in patients in warfarin group at 30 days (*p* = 0.0484), at 180 days (*p* = 0.0005), and at 1 year (*p* < 0.0001). No statistical difference was observed regarding type of fracture (according to AO/OTA classification) and type of surgery (joint reconstruction or fixation with intra- or extramedullary devices).

**Table 1:** Demographic, clinical, and surgery-related characteristics of all patients

	Warfarin	Antiplatelet	Control	Total	*p* value
Patient	157 (12%)	442 (34%)	693 (54%)	1292 (100%)	
Gender					
Male	59 (38%)	115 (26%)	159 (23%)	333 (26%)	0.001
Female	98 (62%)	327 (74%)	534 (77%)	959 (74%)	
Age, mean ± sd	84.76 ± 6.08	84.09 ± 6.61	83.69 ± 8.15	83.96 ± 7.42	0.3567
ASA score					
1/2	0	13 (3%)	69 (10%)	82 (7%)	< 0.001
3	34 (22%)	165 (38%)	350 (51%)	549 (43%)	
4/5	120 (78%)	256 (59%)	265 (39%)	641 (50%)	
CCI, median (IQR, Q1 to Q3)	6 (5–7)	6 (4–7)	5 (4–6)	5 (4–7)	0.0001
AO/OTA Classification					
A1	42 (27%)	93 (21%)	139 (20%)	274 (21%)	0.47
A2	34 (22%)	108 (25%)	164 (24%)	306 (24%)	
A3	7 (4%)	43 (10%)	65 (10%)	115 (9%)	
B1	20 (13%)	70 (16%)	112 (16%)	202 (16%)	
B2	43 (28%)	103 (23%)	168 (24%)	314 (24%)	
B3	10 (6%)	22 (5%)	43 (6%)	75 (6%)	
Types of surgical procedure					
Reconstruction	63 (40%)	178 (40%)	282 (41%)	523 (40%)	0.986
Fixation	94 (60%)	264 (60%)	411 (59%)	769 (60%)	
Time to surgery					
< 48 h	72 (46%)	277 (63%)	479 (69%)	828 (64%)	< 0.001
> 48 h	85 (54%)	165 (37%)	214 (31%)	464 (36%)	
Blood loss (ml)					
Median (IQR, Q1 to Q3)	964.04 (446.95–1652.62)	904.105 (454.4–1486.93)	864.3 (394.59–1349.29)	879.02 (419.33–1446.43)	0.0054
0–490 ml	40 (26%)	119 (27%)	241 (35%)	400 (31%)	0.033
491–980 ml	40 (26%)	139 (31%)	197 (28%)	376 (29%)	
981–1470 ml	30 (19%)	69 (16%)	96 (14%)	195 (15%)	
> 1471 ml	45 (29%)	115 (26%)	157 (23%)	317 (25%)	
Mortality					
30 days	13 (8%)	14 (3%)	28 (4%)	55 (4%)	0.0484*
180 days	42 (27%)	69 (16%)	100 (14%)	211 (16%)	0.0005*
1 year	61 (39%)	109 (25%)	140 (20%)	310 (24%)	< 0.0001*

*Note*: *the *p* values related to mortality at 30 days, 180 days, and 1 year were calculated with the log-rank test

**Fig. 2:**
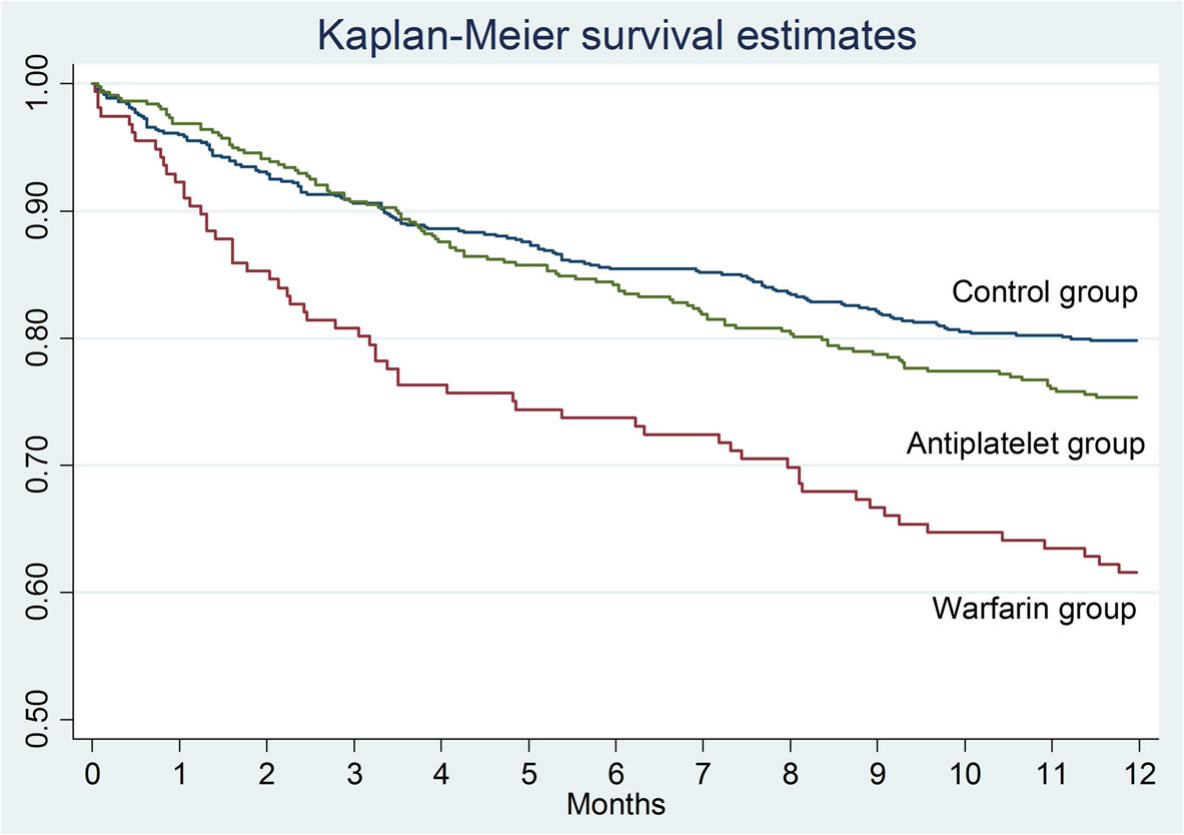
The survival curves show the effect of warfarin treatment status on survival

**Fig. 3:**
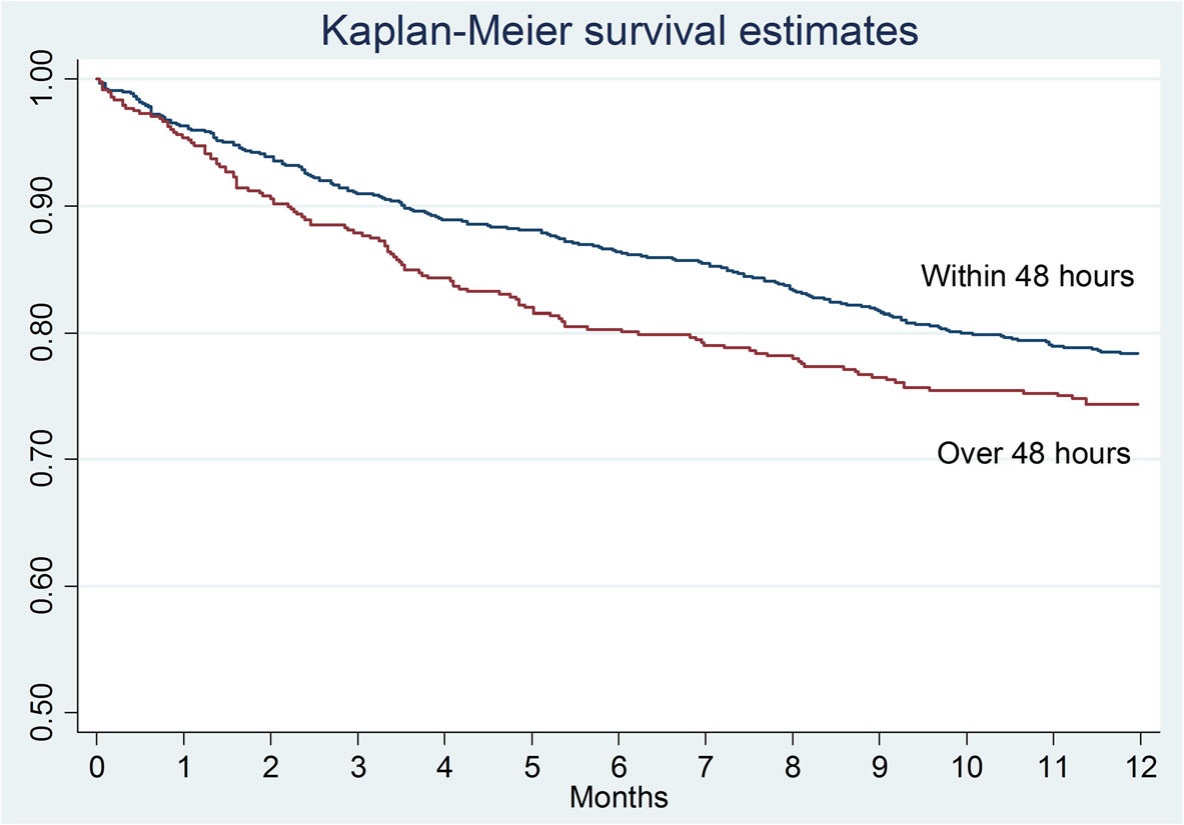
The survival curves show the effect of surgical delay on survival

**Fig. 4:**
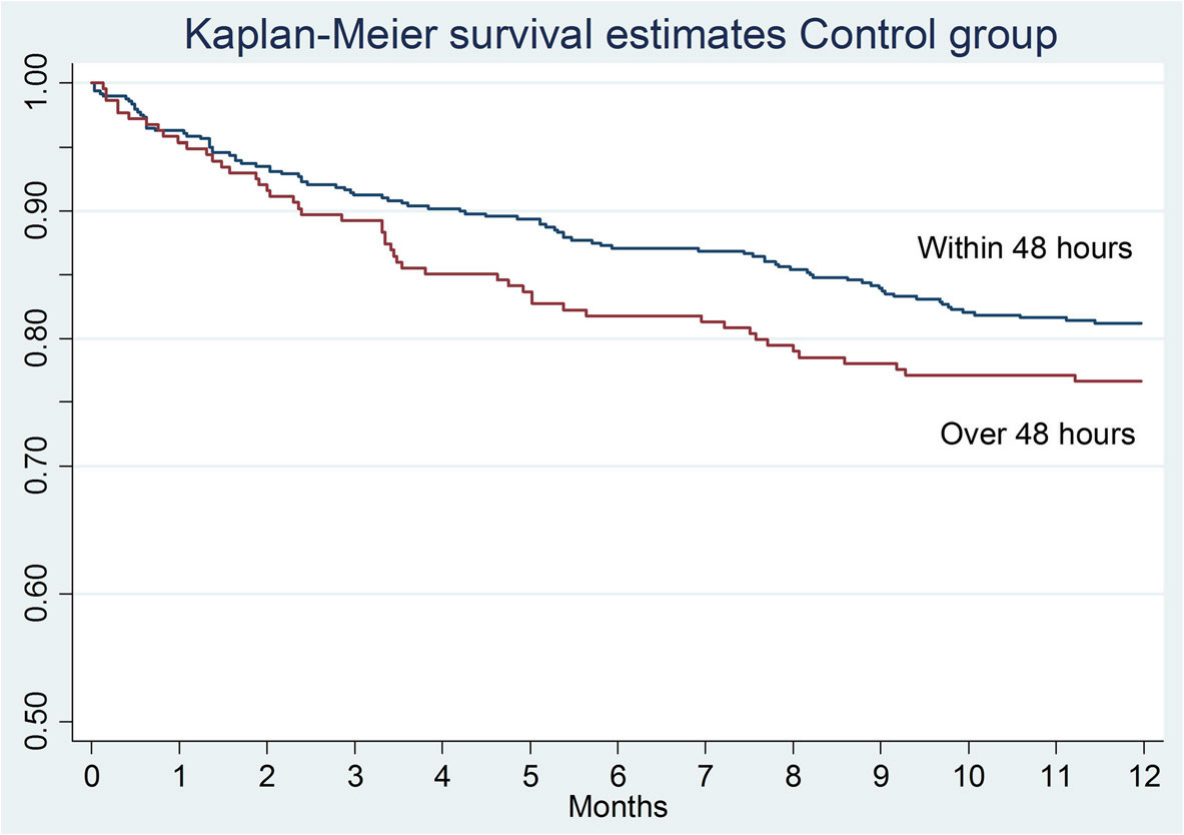
The survival curves show the effect of surgical delay on survival in the control group

**Fig. 5:**
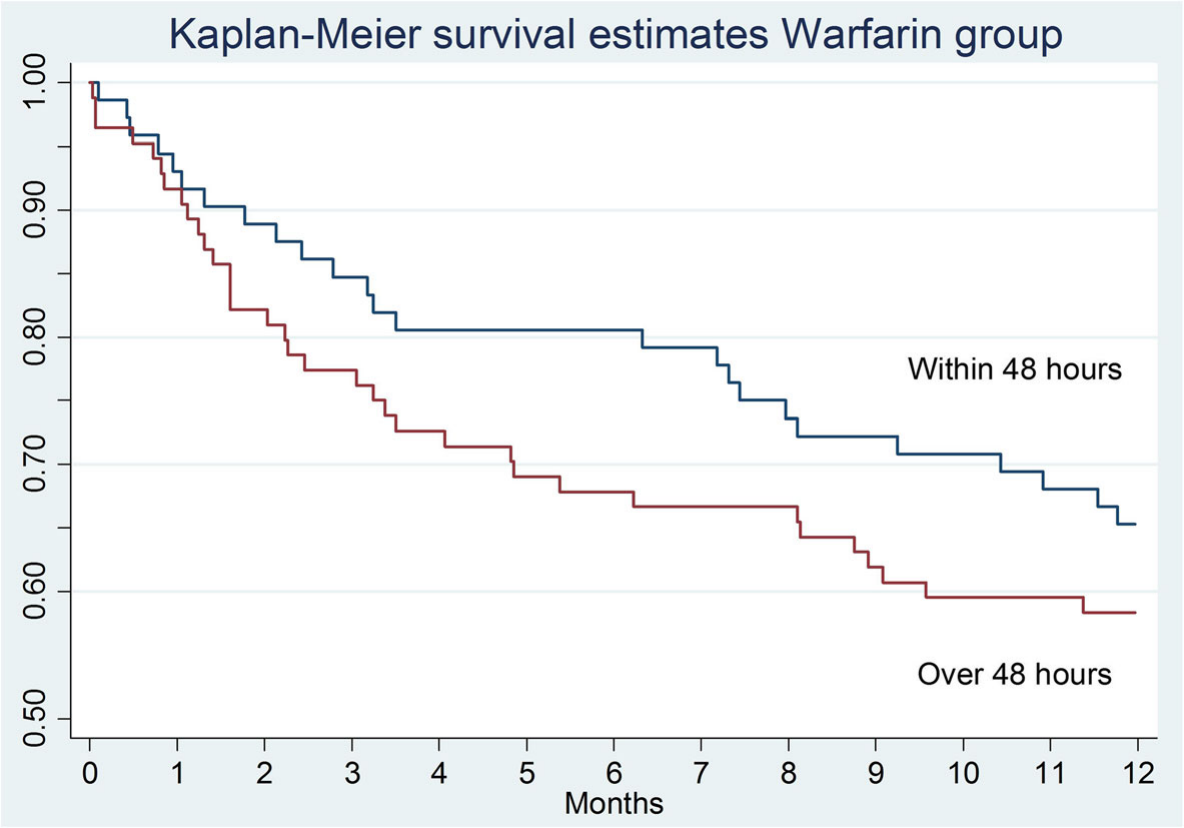
The survival curves show the effect of surgical delay on survival in the warfarin group

The Kaplan–Meier survival curve confirms that mortality of the patients in the warfarin group was significantly higher compared to the other groups (Table 1 and Fig. 2). In terms of the effect of the surgical delay on the survival rate, there are no significant differences in the total sample analyzed (*p* = 0.0654), in the control group (*p* = 0.1440), and in the warfarin one (*p* = 0.3034). Analyzing the Kaplan–Meier survival curves, there is a higher survival trend in patients who underwent surgery within 48 h (Fig. 3) but it is not statistically significant. The same consideration can be made for the control group (Fig. 4) and for the warfarin group (Fig. 5).

Cox multivariate regression analysis showed an increased risk of mortality in patients on warfarin therapy (Table 2), in particular, patients in warfarin therapy have a 42% higher risk of death within 1 year from surgery compared to a patient of the Control group (HRadj = 1.42; CI 95% 1.03–1.95). The patients of the antiplatelet group did not show any significant risk (HRadj = 0.95; CI 95% 0.73–1.23). The patients over 95 years who underwent surgery for a proximal femoral fracture have approximately 3 times higher risk of dying within 1 year compared to patients younger than 75 (HRadj = 2.96; CI 95% 1.65–5.31). As expected, the comorbidities number negatively influenced the survival of these patients. The risk of mortality increases of 24% for every single value of the CCI (HRadj = 1.24; CI 95% 1.17–1.32). Regarding time of surgery, patients who underwent surgery after 48 h have 1.5 times higher risk of mortality with respect to patients who underwent surgery within 48 h, with equal other values (HRadj = 1.53; CI 95% 1.04–2.25).

**Table 2:** Association between baseline patient characteristics and mortality according to Cox regression analysis, unadjusted and adjusted for potential confounders

	Unadjusted Cox regression model			Adjusted Cox regression model		
	Crude HR	CI 95%	*p* value	Adjusted HR	CI 95%	*p* value
Age (ref: 65–74 yrs)						
75–84 yrs	1.13	0.69–1.84	0.633	0.88	0.53–1.45	0.621
85–94 yrs	2.27	1.43–3.61	0.001	1.48	0.92–2.39	0.107
≥ 95 yrs	4.33	2.47–7.57	< 0.001	2.96	1.65–5.31	< 0.001
Gender (ref: male)						
Female	0.63	0.50–0.80	< 0.001	0.71	0.55–0.90	0.005
Therapy (ref: control group)						
Warfarin	2.15	1.59–2.92	< 0.001	1.42	1.03–1.95	0.034
Antiplatelet	1.23	0.96–1.58	0.10	0.95	0.73–1.23	0.687
ASA score (ref: 1/2)						
3	3.27	1.20–8.91	0.021	1.72	0.62–4.79	0.300
4/5	7.79	2.90–20.96	< 0.001	2.46	0.87–6.92	0.089
CCI	1.32	1.26–1.39	< 0.001	1.24	1.17–1.32	< 0.001
Time to surgery (ref: < 48 h)						
> 48 h	1.24	0.99–1.56	0.066	1.53	1.04–2.25	0.032
Blood loss (ref: 0–490 ml)						
491–980 ml	1.92	1.38–2.66	< 0.001	1.60	1.14–2.23	0.006
981–1470 ml	1.97	1.36–2.88	< 0.001	1.38	0.94–2.04	0.097
> 147 ml	2.60	1.88–3.59	< 0.001	1.81	1.30–2.53	< 0.001
AO/OTA Classification (ref: lateral fracture—A)						
Medial fracture—B	0.89	0.71–1.12	0.332			
Types of surgical Procedure (ref: fixation)						
Reconstruction	0.93	0.74–1.17	0.515			

Patients who have a blood loss greater than 1471 ml have a risk of death that is almost twice as high as those who have a blood loss lower than 491 ml (HRadj = 1.81; CI 95% 1.30–2.53).

## Discussion

Nowadays, most recent guidelines underline the importance of early surgery in elderly patients with PFF. However, most of these patients present a high number of comorbidities, some of which require the use of warfarin. These comorbidities and waiting time to achieve a safe INR could cause a surgical delay. We found that warfarinised patients showed higher surgical delay and decreased survival.

Current guidelines suggest to operate these patients when INR values are < 1.5 [13]. Different protocols have been proposed to reach these INR values as soon as possible by using Vitamin K agonists and derivatives, and it has demonstrated that they are safe procedures [23]. Mattison et al. in a recent case-control study analyzed 198 patients with extracapsular PFF and reported that using Vit.K and/or four-factor prothrombin complex concentrate to reach INR values ≤ 1.5 in patients on warfarin therapy is safe [24]. Despite this, Bhatia et al. reported a time to surgery of at least 2 days (mean 38 hrs) in the warfarin group [25]. A study by Ryan et al. showed that surgical delay of more than 48 h resulted in higher mortality in patients with PFF, with an OR of 1.13 for the delayed group after controlling for multiple prognostic factors including comorbidity [26].

Even in our study, warfarinised patients showed higher mortality compared to patients that do not assume any anticoagulant drugs or antiplatelet therapy. In an observational study by Dettoni et al. published in 2011 including 875 patients with PFF, they noticed that warfarin therapy and delay in time to surgery were associated with a significantly higher risk for complications and mortality compared to all other patients [27]. Lawrence et al. showed an association between warfarin therapy with prolonged time to surgery and an increased 1-year mortality rate in hip fracture patients and they suggested to consider warfarin therapy a “Red flag” in patients with PFF [28]. Rosso et al. found out a 365-day mortality of 15.4% in patients who underwent surgery within 48 h, 18.1% between 48 and 72 h, and 21% over 72 h [29]. Recently, Moores et al. [30], in a retrospective analysis of prospectively collected matched data, evaluate post-operative mortality and lengh of stay in hospital for warfarinised hip fracture patients and compare them with a matched nonwarfarinised group, before and after the introduction of a standardized warfarin reversal protocol. They observed that the time of the somministration of the first dose of vitamin K is crucial for the delay in warfarin reversal. They notice that somministration of the first dose decreases from 13 to 3 h (*p* < 0.001) after protocol introduction, with all patients’ INR being in a safe operative range within 24 h of admission.

Furthermore, in our study, we observed that the warfarin patient group had a greater blood loss than the other groups. This result differs from what is reported in Literature [24, 25, 31, 32].

Mattison et al. reported that there were no significant differences in terms of blood loss (*p* = 0.5); meanwhile, the number of units of red blood cell transfused was higher in the non-warfarin group (*p* = 0.03) [24]. Cohn et al. in their study showed that 58.1% of the patients in warfarin underwent transfusion after surgery comparing to a 56.6% in the control group (*p* = 0.86). Also, the blood loss was similar in the two groups (*p* = 0.95) [33]. Even Lott et al. in their study of 479 patients with PFF, in which 112 were in therapy with warfarin, did not notice statistical differences in the two groups in terms of blood loss (*p* = 0.35) and post-operative transfusions (*p* = 0.535) [34].

For these reasons, the PFF NICE guidelines recommend to perform surgery “…on the day of, or the day after admission…” [35]. It is strongly indicated to identify and treat correctable comorbidities that may delay surgery, including anticoagulation therapy. Proactive anticoagulant management and expedient surgery reduce morbidity and mortality when managing this surgically challenging subset of hip fracture patients.

The main limitation of this study is the retrospective design. A randomized control trial would be more suitable to investigate this topic. However, the current knowledge and the guidelines would hardly ethically allow a study that compares early and late surgery in these patients. For this reason, we cannot be certain that awaiting INR was the only factor leading to delay of surgery and we were also unable to obtain specific data on post-operative complications including wound issues, thrombotic events, or pressure sores and causes of death. Additionally, we know the ASA grade, which reflects the comorbidities and the probability of medical complications, but we do not know precisely what these comorbidities are. Therefore, both the antagonization of the warfarin and the investigation and stabilization of these complications could have caused the surgical delay. Furthermore, due to the lack of a standardized institutional protocol, the decision to use warfarin inversion agents at variable times or to wait for INR normalization has the potential to bias. Despite these limitations, our results are consistent with previously published literature.

## Conclusions

Our study confirmed the association between warfarin therapy at the time of admission for hip fracture, time to surgery, and survival rate. Despite the use of vitamin K and four-factor prothrombin complex for a fast reversal of the warfarin effect, waiting for INR values ≤ 1.5 leads to a surgical delay. Surgical delay and comorbidities are the main reasons of the highest mortality of these fragile patients, the only possibility to increase survival rate of these patients is to identify and treat correctable comorbidities and to normalize INR value as soon as possible. For this reason, it is important to implement protocols that are finalized to decrease INR values for a faster surgery, starting from the admission of the patients with PFF on warfarin therapy in the Emergency Department.

## Abbreviations

AO/OTA: AO-Müller/Orthopaedic Trauma Association fracture and dislocation classification
ASA: Society of Anesthesiologists Physical Status Classification System score
CCI: Charlson Comorbity Index
CI: Confidence interval
HR: Hazard ratio
INR: International normalized ratio
PFF: Proximal femoral fracture
SD: Standard deviation
